# Incorporating metadata in HIV transmission network reconstruction: A machine learning feasibility assessment

**DOI:** 10.1371/journal.pcbi.1009336

**Published:** 2021-09-22

**Authors:** Sepideh Mazrouee, Susan J. Little, Joel O. Wertheim

**Affiliations:** Department of Medicine, Division of Infectious Diseases and Global Public Health, University of California San Diego, San Diego, California, United States; Heidelberg University, GERMANY

## Abstract

HIV molecular epidemiology estimates the transmission patterns from clustering genetically similar viruses. The process involves connecting genetically similar genotyped viral sequences in the network implying epidemiological transmissions. This technique relies on genotype data which is collected only from HIV diagnosed and in-care populations and leaves many persons with HIV (PWH) who have no access to consistent care out of the tracking process. We use machine learning algorithms to learn the non-linear correlation patterns between patient metadata and transmissions between HIV-positive cases. This enables us to expand the transmission network reconstruction beyond the molecular network. We employed multiple commonly used supervised classification algorithms to analyze the San Diego Primary Infection Resource Consortium (PIRC) cohort dataset, consisting of genotypes and nearly 80 additional non-genetic features. First, we trained classification models to determine genetically unrelated individuals from related ones. Our results show that random forest and decision tree achieved over 80% in accuracy, precision, recall, and F1-score by only using a subset of meta-features including age, birth sex, sexual orientation, race, transmission category, estimated date of infection, and first viral load date besides genetic data. Additionally, both algorithms achieved approximately 80% sensitivity and specificity. The Area Under Curve (AUC) is reported 97% and 94% for random forest and decision tree classifiers respectively. Next, we extended the models to identify clusters of similar viral sequences. Support vector machine demonstrated one order of magnitude improvement in accuracy of assigning the sequences to the correct cluster compared to dummy uniform random classifier. These results confirm that metadata carries important information about the dynamics of HIV transmission as embedded in transmission clusters. Hence, novel computational approaches are needed to apply the non-trivial knowledge collected from inter-individual genetic information to metadata from PWH in order to expand the estimated transmissions. We note that feature extraction alone will not be effective in identifying patterns of transmission and will result in random clustering of the data, but its utilization in conjunction with genetic data and the right algorithm can contribute to the expansion of the reconstructed network beyond individuals with genetic data.

## 1 Author summary

Molecular transmission networks are built by connecting similar HIV-1 drug resistance viral genomes. Using such methods, approximately half of all sequences stay unlinked, and the remaining half fall into categories of many small clusters and a few large clusters showing fragmentary epidemiological relations. However, the unavailability of genetic data for the entire underlying transmission network and challenges of sampling completeness limits the reliance on creating transmission clusters. Here, we take the known relationship from genetic data and transform the problem into a hybrid multi-step unsupervised and supervised learning problem in which we use contextual data to create a model unwrapping the hidden epidemiological relation among positive cases in transmission networks beyond genetic data. The goal is not to provide a static feature set from metadata that can be used for all jurisdictions but can offer a dynamic classifier framework that can reveal the unique dynamics of the spread of HIV in each jurisdiction.

## 2 Introduction

Observations of closely related viral strains indicate that HIV transmission is occurring rapidly within a common network. Immediately following transmission of HIV between two people, the molecular sequence of the HIV strain in the recipient will be nearly identical to strains found in the transmitting person. As time passes, however, the strains infecting each person will change independently of one another and will look more different [1]. After a diagnosis of HIV for an individual, a sample of their viral sequence is collected for drug resistance purposes and reported to state and local health departments. Health departments together with CDC utilize these drug resistance genotypes for the detection of molecular clusters of epidemiologically related infection incidents. They conduct routine analyses to identify molecular clusters that are concerning for recent and rapid transmission of HIV and probable future growth [2]. The recent CDC guidelines require their funded jurisdictions to run a monthly analysis of the molecular cluster detection using HIV-TRACE [2, 3].

**Fig 1:**
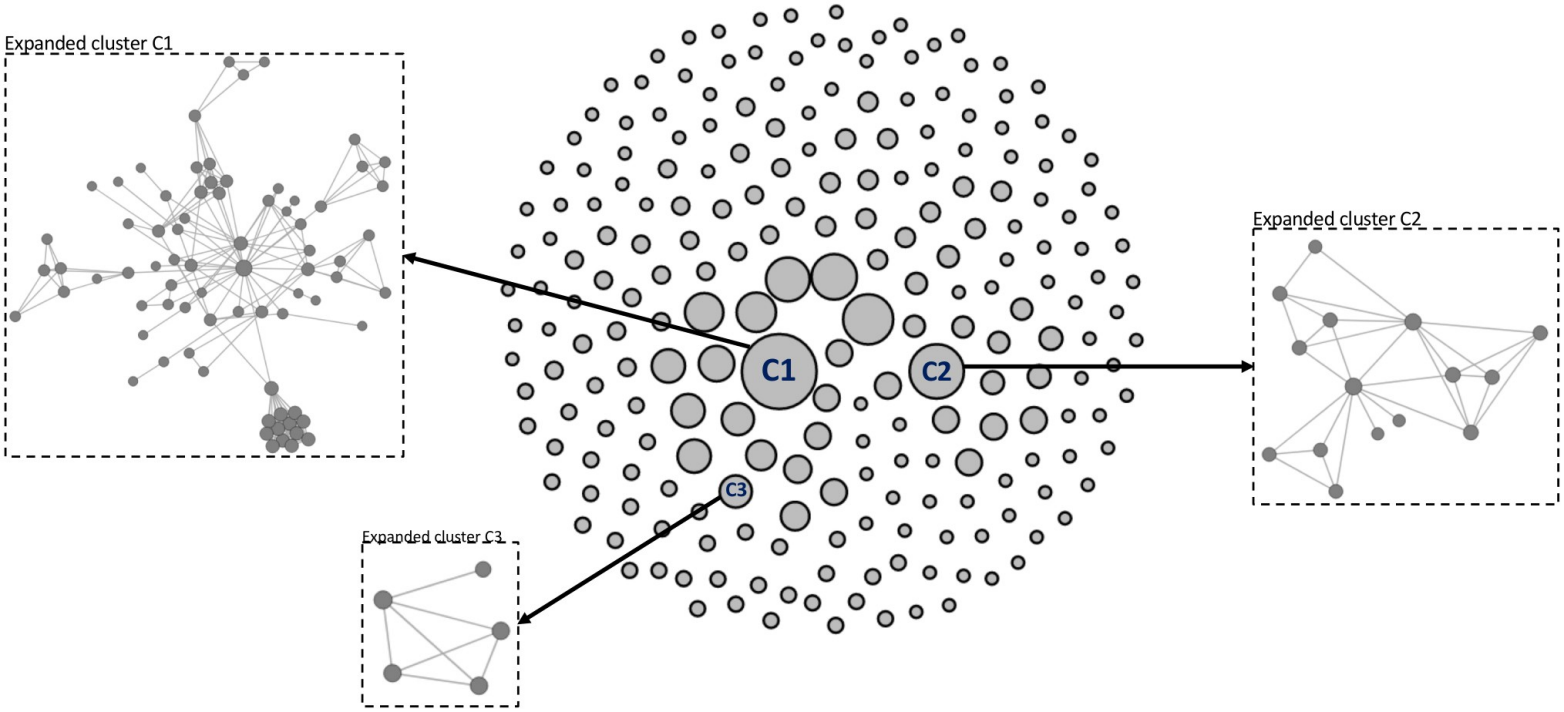
Sample molecular transmission network. Clusters [shown as gray circles with black borderline such as C1, C2 and C3], and three clusters are expanded for more clarity, the nodes represent viral sequences and the edges between every pair of nodes show epidemiological relatedness.

A sample of such reconstructed molecular network is shown in Fig 1. The entire network is split among clusters of related viral strains [shown as gray circles with black borderlines], in which “nodes” represent HIV-1 protease and partial reverse transcriptase genotype sequences and “edges” demonstrate the genetic distance between viral strains (only among the nodes of close proximity to HXB2 reference sequence). The edge weights (pairwise genetic distances) are measured using the TN93 model [4]. After computing all pairwise distances, the method presented in [3] generates a graph of sequence nodes where an edge between two nodes exists if their pairwise distance is ≤ *α*. The *α* is a distance threshold for genetic similarity defined experimentally by field experts, typically between 0.5-2% substitution per site [1, 3]. All connected nodes form a cluster implying epidemiological relatedness. Note that the small genetic distance between two nodes in the graph only indicates that there is an epidemiological connection between two individuals, but cannot provide information regarding the transmission direction. HIV-TRACE groups ≥ 2 nodes together to form clusters of epidemiologically related individuals [5]. The resulting network created with the genetic information identifies genetic links with implied epidemiological links which is a subset of the transmission networks and is interchangeably referred to as transmission networks in literature.

The dynamics of HIV transmission vary in different jurisdictions and prior studies of the network connectivity conform to a scale-free distribution [5]. Usage of molecular clusters to reconstruct molecular transmission networks has revolutionized research on detecting priority clusters and enhanced preventive interventions [6–11]. Although it is a renowned approach, faces limitations that can reduce reliance on genetic sources alone for making inferences about HIV transmission. A large limiting factor is the unavailability of genotype data for the aforementioned transmission and risk networks. To date, the viral genetic sequences collected for drug resistance tests are partly available for people who are living with HIV, while contextual metadata is more accessible for everyone in molecular, transmission, and partial risk networks.

**Fig 2:**
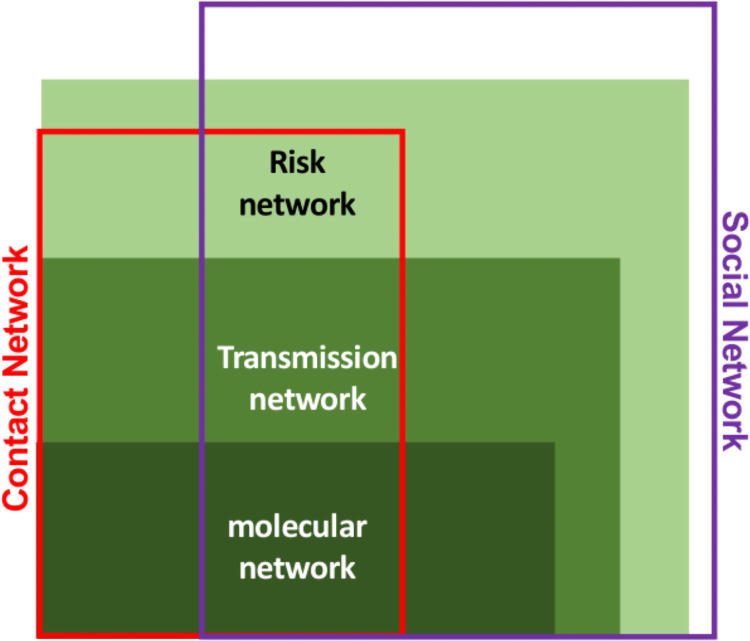
Relationship of the active elements in progressing HIV transmissions.

The metadata (epidemiologic, serologic, behavioral, risk partners) of the individuals in the molecular network have been effectively analyzed to track the origin of foregoing outbreaks and to evaluate transmission dynamics [12–14] for acute and unreported HIV transmissions and to design potent prevention intervention strategies [15, 16]. These findings reveal the importance of information inherent in the metadata associated with the molecular network. Since the dynamics of transmission in a jurisdiction is consistent in the network (regardless of the availability of genetic data), we hypothesize that machine learning algorithms are able to learn these relations from the reconstructed molecular network, built on a strong but limited data source, and apply the acquired knowledge to the metadata in order to expand the estimated network from “molecular” to “transmission” network (color-coded orange and yellow in Fig 2). Here we develop a hybrid multi-step unsupervised and supervised learning model in which a labeled dataset generated from a genetic molecular network trains a classifier to build a model using their associated metadata. The trained algorithm applies the model to the metadata from people without genetic data in the jurisdiction and expands the reconstruction of the transmission networks beyond the molecular networks. This method has the potentials to incorporate more relevant data into the estimation of prior transmissions and might reveal the hidden relationships for the part of singleton nodes that remained unexplored due to the challenges of sampling incompleteness of genomic data. Also, it enables us to refine the chosen genetic distance threshold that might unnecessarily sacrifice links representing the early infections that could be phylogenetically and epidemiologically linked to the outbreak [12, 17].

## 3 Dataset

**Fig 3:**
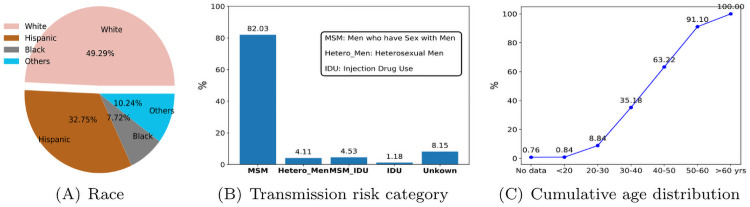
People living with HIV, San Diego PIRC. A. Racial distribution, B. Category of transmission risk, and C. Age distribution.

We analyze the genetic and epidemiological metadata gathered between 1998 and 2019 from 1192 people living with HIV in San Diego Primary Infection Resource Consortium (PIRC). Individuals are clustered together with the genetic distance of ≤0.015 substitutions site. 568 individuals resided in 171 (cluster sizes 2-23). Approximately 47% of all individuals in this study were labeled as clustered and the rest remained as singletons (no evidence of epidemiological relation). PIRC cohort dataset includes nearly 80 demographic, baseline history, laboratory, and screening features. After filtering missing values and performing backward feature elimination, we extracted a small subset of features with the lowest data missingness and high predictive values to utilize as the contextual feature space. Hence, the non-genetic metadata utilized to train the classification algorithms include age, birth sex, sexual orientation, race, transmission category, estimated date of infection, and first viral load date. The racial, transmission category and age distribution of the data is shown in Fig 3 and more in Table 1.

**Table 1:** San Diego PIRC (Primary Infection Resource Consortium) data: 1998-2019.

Variables	All Seqs	Clustered Seqs
**Sexual orientation**		
M	93.53%	97.07%
F	5.54%	2.62%
MF	0.25%	0.31%
FM	0%	0%
Others	0.68%	0%
**First viral load date**		
before 2000	4.7%	6.38%
2000—2005	20.99%	23.43%
2005—2010	12.76%	27.79%
2010—2015	14.17%	28.46%
2015—2019	5.48%	13.94%
Unspecified	41.90%	0%

## 4 Results

In the absence of a gold standard for transmission network, we utilize HIV-TRACE [18] as the baseline to evaluate the effectiveness of our approach. The dataset explained in Section 3, includes genetic and contextual metadata for all individuals. To test our hybrid model we removed the genetic data of the individuals that were used as test data and assumed we only have metadata for those individuals. Then we compared the results of our trained classifiers against HIV-TRACE generated clustered/singletons on the entire dataset as a baseline.

We explored the effect of consolidating the genetic information with metadata to expand the reconstructed transmission network. We first trained a binary-class model using k-nearest neighbor, decision tree, random forest, and support vector machine classifiers trained with contextual metadata explained in Section 3. As shown in Table 2 among all classifiers decision tree and random forest trained using labeled data, gained highest performances in differentiating clustered individuals from singletons using metadata with over 80% in accuracy, precision, recall, and F1-score.

**Table 2:** Macro average performance measures of machine learning algorithms on extended feature set trained classifiers using San Diego cohort data (1998-2019).

Algorithm	Accuracy	Precision	Recall	F1-Score
Decision Tree	0.91	0.89	0.82	0.84
Random Forest	0.86	0.91	0.87	0.88
k-Nearest Neighbors	0.73	0.36	0.50	0.42
Support Vector Machine	0.75	0.71	0.58	0.61

**Fig 4:**
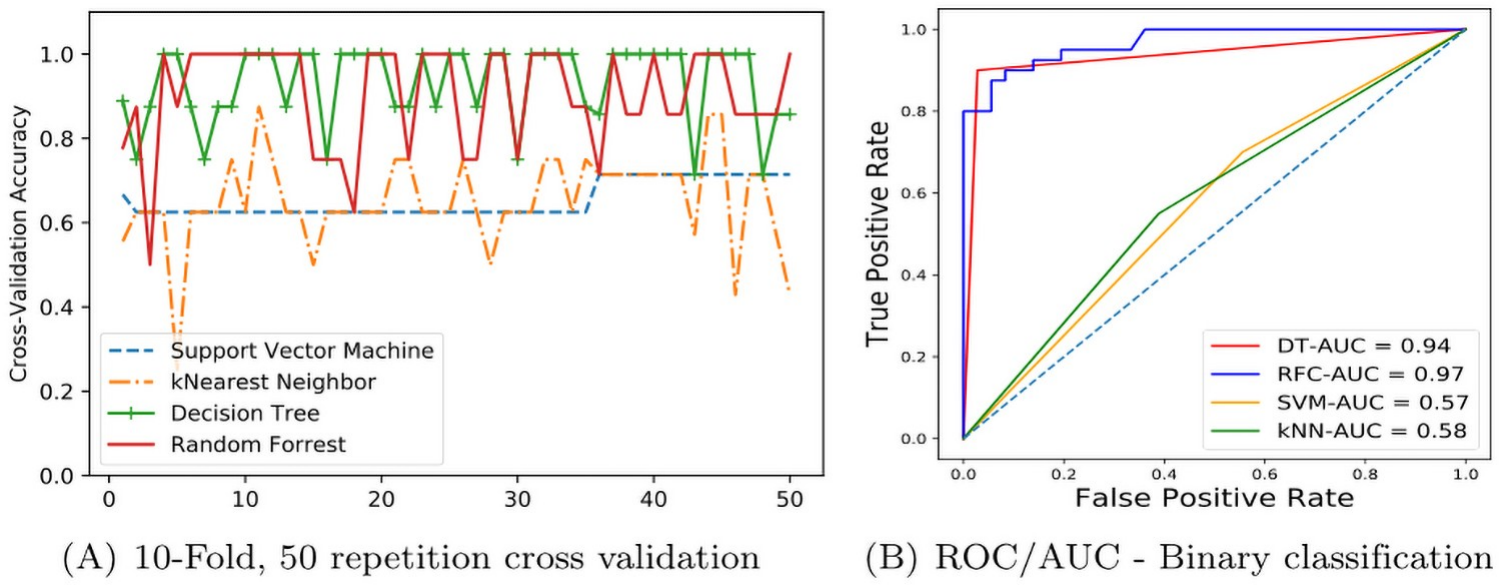
Accuracy measures of the trained models. A. Cross validation—binary classification, B. ROC and Area Under Curve.

To assure independence in k-fold cross validation training, we performed the cross-validation with 50 repetitions, to avoid any possible estimation bias. Consistent with the train-test model results [in Table 2], cross-validation accuracy reports decision tree and random forest achieving highest performance in most repetitions [shown in Fig 4A]. We also generated Receiver Operating Characteristic (ROC) curves for the algorithms and calculated the Area Under Curve (AUC) shown in Fig 4B is reported 97% and 94% for random forest and decision tree classifiers respectively. We computed other accuracy measures such as the sensitivity and specificity of each algorithm. The random forest and decision tree achieved over 90% sensitivity and 80% specificity [shown in Table 3] which confirms the performance measures achieved using test-train or cross-validation learning.

**Table 3:** Predictive performance measures.

Algorithm	Sensitivity	Specificity
Decision Tree	0.94	0.9
Random Forest	0.91	0.82
k-Nearest Neighbor	0.45	0.77
Support Vector Machine	0.47	0.70

**Fig 5:**
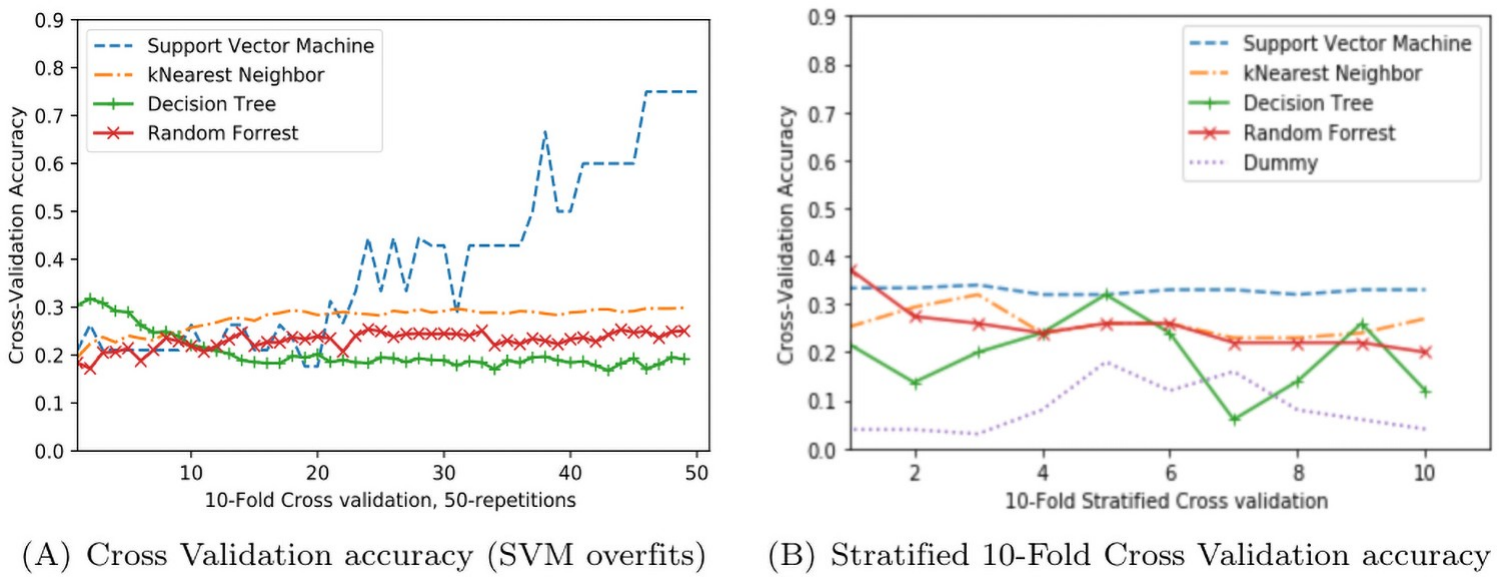
Accuracy comparison for node assignments to 29-clusters incorporating metadata. A. Cross validation, B. Stratified Cross Validation.

The performance results for a binary classification (of clustered vs. singletons) is demonstrated in Tables 2 and 3, and Fig 4. To test the extended hypothesis from binary classification differentiating among clusters, we trained models with clusters of size ≥ 5 using 50 repetitions of 10-Fold cross-validation. Despite choosing a high value for K the evaluations of the support vector machine in some repetitions raised overfitting concerns [see Fig 5A]. Therefore, to detect overfitting of the classifier we performed stratified cross-validation in that we rearrange the data whereby each fold has a good representation of the whole dataset. The accuracy achieved by SVM outperformed other classifiers [Fig 5B]. Although approximately 0.30% accuracy seems modest, please note that the accuracy for Dummy Uniform random classifier in a 30-class classification problem is also demonstrated as the baseline performance. The results show that the SVM prediction class assignment approach is on average an order of magnitude better than the random classifier.

Example code providing an implementation of the the method is available at: https://github.com/smazrouee/MLMetadata. Patient level metadata cannot be shared publicaly due to risk of violating privacy. HIV-1 sequence data is available at: https://www.ncbi.nlm.nih.gov/genome/viruses/retroviruses [18, 19].

## 5 Discussion

The development of efficient and scalable algorithms for the reconstruction of transmission networks remains an open research problem. Quality of viral strains, sequence alignment specifications, complications in sampling molecular and epidemiological data, and the density and relevance of the variables to explain transmissions make the problem challenging. Current methods are built based on viral genetic data not utilizing metadata which carries valuable information for the reconstruction of transmission networks. We presented a framework to enhance our ability to incorporate useful information from contextual metadata. Our approach extracts knowledge from HIV-1 drug resistance genetic data and reflects it on demographic, transmission risk, laboratory, comorbidity, and social network data in order to provide a more comprehensive view of transmission dynamics of the disease. We examined if the machine learning algorithms can identify similar epidemiologically related groups of individuals and comparing machine-learning-based prediction results to those of a baseline approach in HIV-TRACE. The performance results of classification models demonstrate promising results that a trained classifier is beneficial in recognizing epidemiological relations. Using such models the estimated networks become closer to the underlying transmission network with a core based on molecular data and the extension parts of the reconstructed network can be estimated more accurately with the expansion of the relevant metadata. Therefore, the prevention interventions will be applied to a larger population at risk rather than the core molecular network. Another recent study also demonstrated the effectiveness of similar machine learning models in identifying clusters with future transmissions in a statewide investigation [20]. Evidently, the density and coherency of the collected data for each feature that varies in different datasets can potentially affect the results of the clustering or classification models. Hence, neither can be a fixed list of metadata variables across all jurisdictions to distinguish relatedness nor a static classifier that can be applied to all jurisdictions. Instead a dynamic classifier should be design that can be trained from local dataset and extract the patterns for proper predictions. Therefore, studying several jurisdictions in different states or countries will be beneficial to build a model that can determine a sufficient feature space that would lead to a reasonable transmission network reconstruction.

Although using a moderate set of extended features resulted in a modest accuracy performance, it validates the feasibility of using a classifier trained with genetic data for distinguishing potential relatedness of a new HIV positive with no genotype which ultimately takes molecular networks one step closer to the underlying transmission network. Application of such models on surveillance data with the larger feature sets and possibly more number of cases could result in more accurate and generalizable algorithms. In general to choose which machine learning algorithm can uncover the dynamics of a network, first we need to have a clear picture of the data, the problem, and the constraints. In the context of the HIV transmission network we should note that only using feature extractions single-handedly will not be effective in the determination of the outbreak patterns and will result in random clustering of the jurisdiction data, but utilization of such data in conjunction with genetic data and the right algorithm.

Overall, our results suggest that metadata contains useful information about transmission dynamics, and machine learning algorithms can be used to find a mapping between metadata and transmission clusters. However, studying larger surveillance datasets comprising far greater feature space not limited to demographic or genetic information can be an extension of this project in which efficiency of neural networks and deep learning on such higher dimension datasets would be beneficial. Expansion of these approaches to larger jurisdiction public health surveillance databases will enhance our ability to predict these incident infections [21, 22].

## 6 Methods

**Fig 6:**
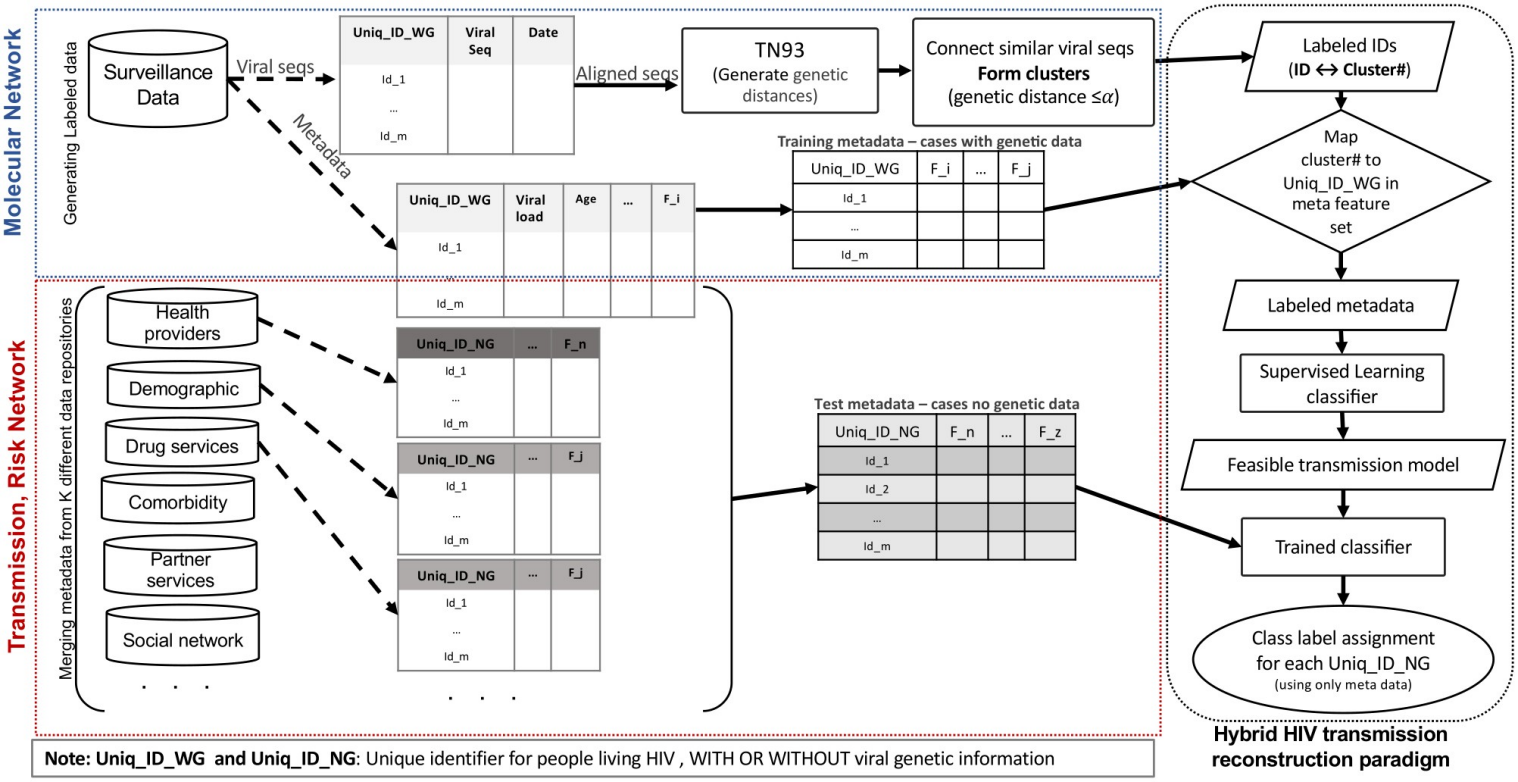
Hybrid unsupervised/supervised HIV transmission reconstruction model pipeline. Generating a labeled dataset from molecular data (blue dashed line box), reconstructing transmission network using labeled dataset (red dashed box), classification using augmented genetic and non genetic data (black dashed box).

The goal of this study is to examine the feasibility of analyzing contextual metadata to expand the reconstruction of HIV transmission beyond the molecular network of people who are living with HIV/AIDS. Such molecular data are collected only from HIV diagnosed and in-care populations, leaving many persons with HIV (PWH) who have no access to consistent care out of the tracking process. Here, we develop a dynamic framework consisting of a hybrid multi-step unsupervised and supervised learning algorithm in which we create a labeled dataset from the viral genetic data and use their contextual metadata (epidemiological, demographic, etc) to create a dynamic model to unwrap the non-random transmission patterns of HIV in the jurisdiction. An overview of the processing pipeline is illustrated in Fig 6. It comprises a two-phase hybrid model in which the first phase generates a labeled training dataset. In the second phase, the labeled dataset will be used by a trained classifier to process the metadata from people living with HIV whose genetic sequence is not available to determine whether they are epidemiologically related to the rest of the network by assigning them to the clustered or the singleton class (or assign them to a unique cluster identifier). We would like to note here that we do not assume that the nodes labeled as singletons are inherently irrelevant to the epidemic of the jurisdiction under study. We used the CDC definition for clusters exhibiting recent and rapid growth for analysis of the PIRC dataset [23], and believe the disconnection of a large portion of genotype data in the molecular network (leaving many as singletons) is related to the data completeness issue presented in a recent study [17]. The higher level of data completeness will introduce less sparsity and more data resolution which indeed improves the performance of the models presented in this study.

The sequences with genetic distance ≥ *α* will be labeled as singletons implying no evidence in the data for epidemiological relation between the corresponding singleton nodes and the rest of the transmission network. Note here that the method presented in [24] is a data-driven heuristic based on the genetic evolution model presented in TN93 which can be impacted by data missingness for cluster detection as shown in [17]. Therefore, no linkage in the estimated transmission network, for a singleton node in one time frame does not mean complete ignorance of that node. Instead, analysis of longitudinal data might close the gap of the missing individual’s data in the forthcoming batches of data. Also note that, the majority of singletons are genetically far from the clustered individuals. Therefore with multiple runs using the same dataset the results will not change unless the genetic threshold or other network configuration is modified. This demonstrates robustness of our cluster identification approach. However, this is a data-dependent problem and the results would be impacted if we had a smaller sample size. Evidently, having more data results in finding more clusters.

We further investigated whether using trained classifiers could be useful not only in distinguishing between clustered nodes vs singletons but also in the possibility of cluster determination. Therefore we trained models using the same classifiers but this time instead of the binary class labels (clustered vs. singleton), we used the cluster unique identifiers as class labels for each clustered sequence [shown in step(5) of Algorithm 1].

### 6.1 Machine learning algorithm design

We first generate a labeled dataset by using clustered or singleton individuals from genetically linked viral sequences of surveillance data. Assume that the dataset has a size of *m* individuals with viral sequences. If their pairwise genetic distances (generated by TN93 [4]) is smaller than a predefined threshold (*α*) the sequences are connected to form a cluster of epidemiologically related individuals. Let $X=\{X_{1},X_{2},\ldots ,X_{m}\}$ be the metadata of size *m* containing data of the *m* individuals. Each instance *X*_*i*_ in X is a vector of *n* meta-features. Therefore, each instance can be written as *X*_*i*_ = [*f*_*i*1_, *f*_*i*2_, …, *f*_*in*_] where *f*_*ij*_ denotes the value of the *j*-the meta feature for the *i*-the individual. Each instance *X*_*i*_ (meta feature vector) in X is assigned a class label of clustered vs singleton for the *i*-the individual. The addition of these class labels to the metadata from these individuals results in obtaining a labeled training dataset, which is then fed into a supervised learning classification algorithm. Here we used decision tree, random forest (120 estimators), k-nearest-neighbors (n: number of clusters), and support vector machine classifiers. The process of training a machine-learning algorithm requires a distance metric for comparing different instances in X. We measure the amount of dissimilarity between each pair of individuals using Euclidean distance (i.e., *L*^1^ norm) in the meta feature space. Therefore, the distance between two individuals with meta instances *X*_*i*_ and *X*_*j*_ is computed as:

$$\begin{array}{c} \Delta_{ij}=\|X_{i}-X_{j}{\|}^{2} \end{array}$$ (1)

First, we evaluated the classifiers using train-test sets (70 to 30 ratio). Having in mind, the size of the dataset under study and the facts shown in Table 1, Fig 3 (that the data is highly skewed towards specific race, sexual orientation, and transmission category), we decided to evaluate the performance of all classifiers using a method which is robust against data imbalance known as k-fold cross-validation. Cross-validation is a resampling procedure that involves randomly dividing the dataset into k groups (or k-fold) of approximately equal size. We performed a sensitivity analysis for K we chose *K* = 10 in which our entire dataset is split into 10. The first fold is treated as a validation set and the method is trained on the remaining 9 folds. In the next fold, another subgroup among 10 split data is held out as the test dataset and the remaining groups as the training set. In every fold, the model is fit using the training (over 1,000 nodes) and evaluated on the testing set (about 120 nodes). The procedure repeats until the last fold among 10-fold. Although 10-fold cross-validation is not susceptible to classification bias, we decided to conduct other methods to preclude any possibility of bias or overfitting. Therefore, we assessed the models again using repetitive and stratified cross-validation. We performed evaluations on all four models to measure whether the trained classifier can automatically assign a new positive case to the class labels it belongs to (clustered class label: potentially related to the molecular network, or singleton class label: no evidence of relatedness to the molecular network). In the Algorithm 1 we describe the step-by-step method.

**Algorithm 1** Hybrid HIV transmission reconstruction model

**Require**: Pairwise genetic distance of viral strains using TN93

 **(1)** Create graph *G* = (*V*,*E*,*W*_*E*_)

 **while** (not all vertices are processed) **do**

  **(2)** If *W*_*E*_ >*α*, label the corresponding nodes (*V*) as **singletons**

  **(3)** Cluster *G* into *C* = [*C*_1_..*C*_*n*_] from all connected components: where *W*_*E*_ ≤ *α* and label the corresponding nodes (*V*) as **clustered**

  **(4)** Report binary labels (**clustered vs. singletons**) for all nodes

  **(5)** Report **cluster ids**: *C* = [*C*_1_..*C*_*n*_] for clustered nodes

 **end while**

 **for** all nodes *V* **do**

  **(6)** List each *V*_*i*_ along with the corresponding binary label (clustered vs singleton)

  **(7)** Incorporate the non-genetic features of *V*_*i*_ besides its binary class label to form metadata feature space

 **end for**

 **(8)** Split data into training and testing sets

 **(9)** Train models using different classifiers to automatically map each infection event onto the clustered or singleton label from step (4)

 **(10)** Validate the accuracy of class assignments using the testing dataset

In addition to binary classification (clustered vs. singletons) which is beneficial in recognizing related infection incidents, we trained classifiers with the genetic information of infected populations to identifying clusters. To test the extended hypothesis from binary classification for cluster identification, we thought clusters size 2 or 3 might not have enough nodes to exhibit an acceptable level of resolution among these clusters. Therefore, we decided to test it with larger clusters of size ≥ 5. San Diego cohort dataset clusters of size ≥ 5 comprise 43.8% of all clustered individuals residing in 29 clusters. We used cross-validation in multiple repetitions and also repeated the evaluation of the cluster identification using stratified cross-validation re-sampling method to avoid bias and overfitting possibilities.
